# Alternative lengthening of telomeres is mechanistically linked to potential therapeutic vulnerability in the stem‐like subtype of gastric cancer

**DOI:** 10.1002/ctm2.561

**Published:** 2021-09-14

**Authors:** Ji‐Yong Sung, Jae‐Ho Cheong

**Affiliations:** ^1^ Department of Biomedical Systems Informatics Yonsei University College of Medicine Seoul Korea; ^2^ Department of Surgery Yonsei University College of Medicine Seoul Korea; ^3^ Yonsei Biomedical Research Institute Yonsei University College of Medicine Seoul Korea; ^4^ Brain Korea 21 PLUS Project for Medical Science Yonsei University College of Medicine Seoul Korea; ^5^ Department of Biochemistry and Molecular Biology Yonsei University College of Medicine Seoul Korea; ^6^ Department of Research & development VeraVerse Inc. Seoul Korea

Dear Editor,

Alternative lengthening of telomeres (ALT) is a telomere maintenance mechanism (TMM) frequently observed in recalcitrant cancer. However, the TMM in the cancer stem‐like subtype of gastric cancer (GC) is unknown. To assess the therapeutic targetability of the TMM, we analyzed transcriptome data of 497 GC patients,^1^ classified into ALT‐like and non‐ALT tumor groups based on chromatin decompaction. Among five GC subtypes (Figure 1A,C), 92.3% of stem‐like subtype samples exhibited high‐level chromatin decompaction. According to currently proposed ALT mechanisms, *ZNF827* recruits the NuRD complex to telomeres. The resultant NuRD‐ZNF827 complex has ALT‐promoting activity.^2^ ALT‐like tumors were detected predominantly in the stem‐like GC subtype (45%), whereas non‐ALT tumors were detected primarily in the inflammatory and intestinal subtypes (both 30%; Figure 1B). Of the 117 stem‐like subtype samples, 108 (92.3%) were classified as ALT‐like tumors (Figure 1D). The five subtypes have different molecular characteristics and prognoses^1^ (Figures S1, S2). Compared with that in other subtypes, telomerase activity was substantially decreased or absent in the stem‐like subtype (Figure S2A). We evaluated 97 TMM‐related genes (Table S1) and determined differentially expressed genes in ALT‐like and non‐ALT tumors (Figure 1F). *NR2F2* (–log *p *= 15.65) and *ZNF827* (–log *p *= 15.65) had significantly higher expression levels in ALT‐like tumors than in non‐ALT tumors. Compared with those in non‐ALT tumors, *ABL1* (–log *p *= 15.65), *ZCCHC7* (–log *p *= 15.65), and *HSPA1A* (–log *p* = 15.65) were overexpressed in ALT‐like tumors (Figure 1G, Table S2). Histone modifiers trigger telomeric chromatin decompaction, thus reducing telomeric chromatin compaction and causing an ALT‐like phenotype.^3^ The highest chromatin group showed low or no telomerase activity in different GC cohorts (Figures 1E and 2A). When stratified by chromatin decompaction, non‐ALT tumors were associated with a better prognosis (*p* = 0.017) than ALT‐like tumors (Figure 1H,I; Table S3). This is consistent with previous findings that low ALT activity is associated with a better prognosis than high ALT activity in most cancer types.^4^

**FIGURE 1:**
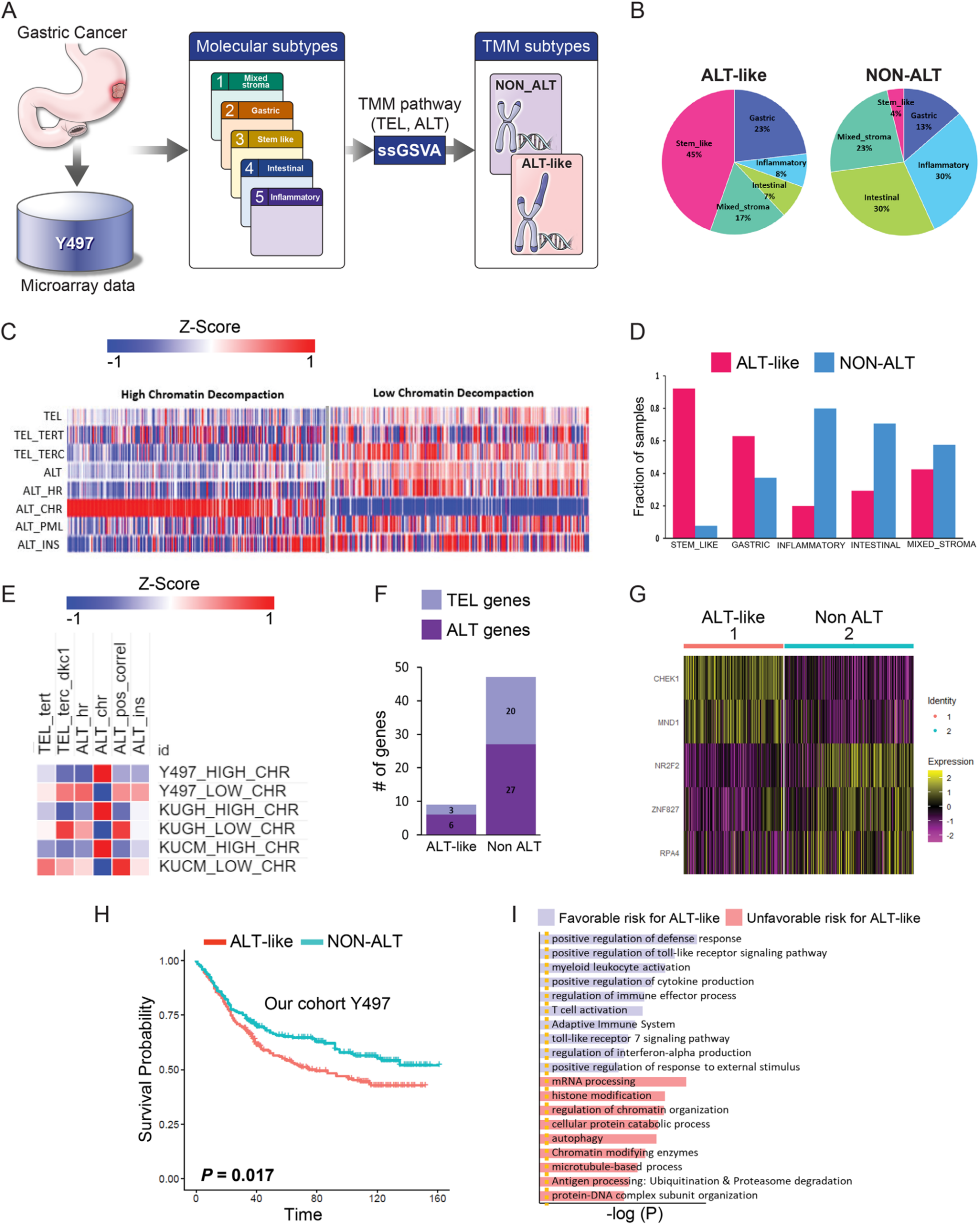
Transcriptional telomere maintenance mechanism (TMM) analysis in gastric cancer (GC). (A) Overview of the analysis pipeline. (B) Pie chart showing the frequency distribution of molecular subtypes in alternative lengthening of telomeres ALT‐like (*n* = 254) and non‐ALT (*n* = 243) tumors: 45% stem‐like, 23% gastric, 8% inflammatory, 7% intestinal, 17% mixed stromal for ALT‐like; 30% inflammatory, 30% intestinal, 23% mixed stromal, 13% gastric, 4% stem‐like for non‐ALT. (C) TMM of ALT‐like and non‐ALT in 497 GC samples. Tel_TERT, telomere TERT pathway; Tel_TERC_DKC1, telomere TERC DKC1 pathway; ALT_HR, ALT homologous recombination pathway; ALT_CHR, ALT chromatin decompaction pathway; ALT_PML, ALT PML pathway; ALT_ins, ALT telomere instability pathway. p‐Values were calculated using a two‐sided Student's t‐test and adjusted for multiple testing (Benjamini‐Hochberg) using FDR correction. p < 0.05 was considered significant. The telomerase pathway consists of the TERT, TERC, and DKC1 pathways, with 38 genes. The ALT pathway includes four sub‐pathways: the homologous recombination (HR) pathway, chromatin decompaction pathway, PML pathway, and TERRA induction and telomere instability pathway, with 59 genes. We calculated eight pathway enrichment scores per sample and repeated the analysis 10,000,000 times to generate the background distribution of significant hits, from which we assessed whether the observed numbers were significantly higher than random expectation. For single‐sample gene set enrichment analysis (ssGSEA), we used the “GSVA” R package. Samples were classified as ALT‐like or non‐ALT based on FDR < 0.5 and chromatin status (“upregulated” or “downregulated”). Through this systematic analysis, each sample was assigned two telomere maintenance subtypes. Hierarchical clustering of log10‐transformed expression data was performed using the one minus Pearson correlation and the metric average method. (D) Bar graph showing the frequency distribution of ALT‐like and non‐ALT tumors according to molecular subtype: red indicates ALT‐like and blue indicates non‐ALT tumors. 92.30% stem‐like, 62.92% gastric, 42.42% mixed stromal, 29.41% intestinal, 20% inflammatory in ALT‐like, 7.69% stem‐like, 37.07% gastric, 80% inflammatory, 70.58% intestinal, and 57.57% for mixed stromal subtype in non‐ALT. (E) Heatmap of TMM in multiple cohorts. (F) Bar graph showing differentially expressed genes in TMM: three TEL genes and six ALT genes for ALT‐like, 20 TEL genes and 27 ALT genes for non‐ALT were significantly differentially expressed. (G) Heatmap of significantly overexpressed genes in ALT‐like versus non‐ALT tumors (H) Kaplan–Meier plots showing overall survival rates for the ALT‐like and non‐ALT groups. *p*‐values were calculated using the log‐rank test. (I) Gene ontology analysis of the ALT‐like and non‐ALT groups for the good patient outcome on non‐ALT in GC. ALT‐enriched biological pathways are indicated in blue and non‐ALT‐enriched biological pathways in red. Gene ontology (GO) describes gene products with three independent categories: biological process, cellular component, and molecular function.

**FIGURE 2:**
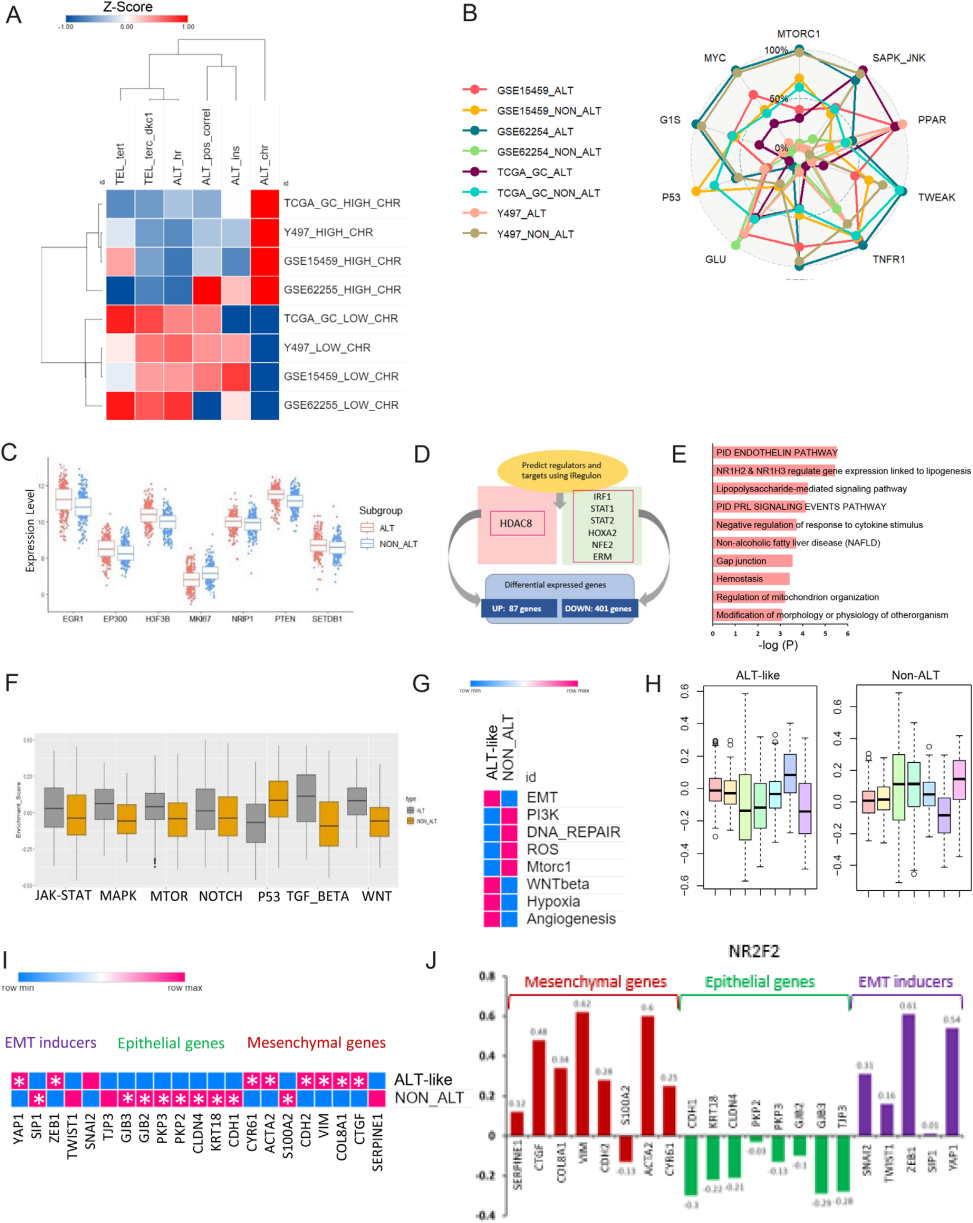
Hallmarks of alternative lengthening of telomeres (ALT)‐like in GC. (A) Heatmap of telomere maintenance mechanism (TMM) in four different cohorts. (B) Spider plot of ALT‐like phenotypes among four cohorts. (C) Box plot of key genes involved in ALT‐like (*p* < 0.05). (D) Regulators in ALT‐like and non‐ALT predicted using iRegulon. Two hundred ninety genes were overexpressed in ALT‐like and 397 genes were overexpressed in non‐ALT tumors. (E) Transcription factor (TF) and targeted gene network for ALT. (F) Box plot for seven cancer signaling pathways in ALT‐like and non‐ALT: p53 (p < 2.22e–16), WNT (*p* < 2.22e–16), NOTCH (*p* = 0.0099), TGF‐BETA (*p* < 2.22e–16), JAK‐STAT (*p* = 0.0079), MAPK (*p* = 9.9e–16), and MTOR (*p* = 2.5e–08). To explore the biological processes associated with the TMM, we analyzed TMM, ALT‐like, and non‐ALT cancer hallmark pathways, including ROS, DNA damage, EMT, hypoxia, angiogenesis, and G2M. The seven selected cancer signaling pathways included p53, WNT, NOTCH, TGF‐BETA, JAK‐STAT, MAPK, mTOR signaling, and the gene sets were obtained from MSigDB (http://software.broadinstitute.org/gsea/msigdb). To evaluate pathway enrichment or depletion, we used ssGSEA in the “GSVA” R package. We calculated eight pathway enrichment scores per sample and repeated the analysis 10,000,000 times to generate the background distribution of significant hits, from which we assessed whether the observed numbers were significantly higher than random expectation. (G) Eight cancer hallmarks (PI3K, Mtorc1, WNT, G2M checkpoint, EMT, DNA repair, p53, ROS, hypoxia, and angiogenesis) and six TMM in ALT‐like and non‐ALT. Significant enrichments (FDR < 0.01) are indicated in pink or blue. Dark brown indicates the enrichment of a hallmark gene set in genes highly expressed in ALT. Blue indicates the opposite pattern. (H) Box plot for seven metabolic signature pathways in ALT‐like and non‐ALT. lipids (red): *p* = 0.1981, carbohydrates (yellow): *p* = 4.267e–07, TCA (yellow green): *p* = 2.442e–16, amino acids (green): *p* < 2.2e–16, vitamins (sky blue): p < 2.2e–16, energy (blue): *p* < 2.2e–16, nucleotides (purple): *p* < 2.2e–16 (I) Heatmap (pink indicates high expression and blue indicates low expression, FDR < 0.05) for EMT signatures: mesenchymal genes, epithelial genes, and EMT inducers. (J) Bar chart of correlations between EMT member genes and *NR2F2* (red: mesenchymal genes, green: epithelial genes, EMT‐inducer genes). Abbreviations: Alternative lengthening of telomeres (ALT); epithelial‐mesenchymal transition (EMT); gastric cancer (GC); and telomere maintenance mechanism (TMM)

Among 10 ALT phenotypes, the Y497 cohort exhibited enhanced PPAR pathway activation and upregulated glucocorticoid receptor expression (Figure 2B). Analysis of the differentially expressed genes related to TMM showed that nine genes were significantly expressed in ALT‐like tumors (Figure 1F, Table S2). *EGR1* had higher expression levels in ALT‐like than in non‐ALT tumors (FDR* =* 0.001; Figure 2C). *EGR1* has dual roles. It acts as an activator of hTERT expression in aggressive cancer cell lines^5^ and as a repressor of hTERT expression, promoting cell proliferation and invasion by upregulating beta‐catenin expression, in GC cell lines.^6^ Using iRegulon, we identified HDAC8 as a transcription factor related to ALT (FDR = 0.001; Figure 2D, Table S4). HDAC8 was found to regulate 87 genes, including NR1H2 and NR1H3, which are involved in regulating lipogenesis‐related gene expression (Figure 2E).

In ALT‐like tumors, heterochromatin was decreased and the p53 pathway activity was low (Figure 2F). Non‐ALT tumors showed a greater extent of DNA repair and higher ROS levels than ALT‐like tumors (Figure 2G).

Epithelial‐mesenchymal transition (EMT) provides a strong link between ALT‐associated PML body formation and telomere extension as well as between ALT activation and mesenchymal tumors.^7^ In ALT‐like tumors, EMT was strongly correlated with chromatin decompaction. EMT‐inducer genes, particularly *ZEB1* (R = 0.61, *p* = 1.21E‐14) and *YAP1* (R = 0.54, *p *= 0.000482), were highly correlated with *NR2F2* (Figure 2I and J). Our results showed an association between ALT‐like tumors and microsatellite instability (MSI) status, which is another characteristic of GC (Figure S3). In the presence of telomere lengthening, ALT pathway activity related to chromatin decompaction was the highest in low MSI samples (*p* < 0.000001); these samples showed a similar TMM pattern to ALT‐like samples with low telomerase and high ALT activities (Figure S3). In the telomere extension samples, high MSI was associated with a better patient prognosis than low MSI. ALT is heterogeneous within cancer samples undergoing telomere extension.^4^ These results indicate that ALT‐like GC tumors are of the mesenchymal type owing to high EMT and likely to be of low MSI status. We analyzed gene sets of seven metabolic pathways based on REACTOME annotation.^8^ Energy‐related genes (*p* < 2.2e–16) were significantly enriched in ALT (Figure 2H). Different metabolic reprogramming was observed in each GC subtype (Figure S1A).

Further, we predicted that the telomere length would be very short because of the high *PML* expression level in non‐ALT tumors compared with that in ALT‐like tumors of the Cancer Genome Atlas Stomach Adenocarcinoma (TCGA STAD) cohort.^4^ Moreover, upregulated expression of *PML* and *KI67* has been observed in colorectal tumors with short telomeres.^9^ Thirty‐nine ALT and 57 mitochondrial biogenesis genes showed positive and negative correlation patterns, respectively (Figure 3A). The overexpression of energy‐related genes in the ALT group was enriched in the “regulation of insulin secretion” (Figure 3B). *CREBBP*, *PPARGC1A*, *NCOA1*, and *MEF2C* were predicted to interact with NR2F2 (Figure 3D). We found that the risk was higher (*p* < 0.001) in ALT‐like samples than in non‐ALT samples (Figure 3C), indicating the clinical relevance of the high chromatin ALT group in energy metabolism, ALT, and EMT.

**FIGURE 3:**
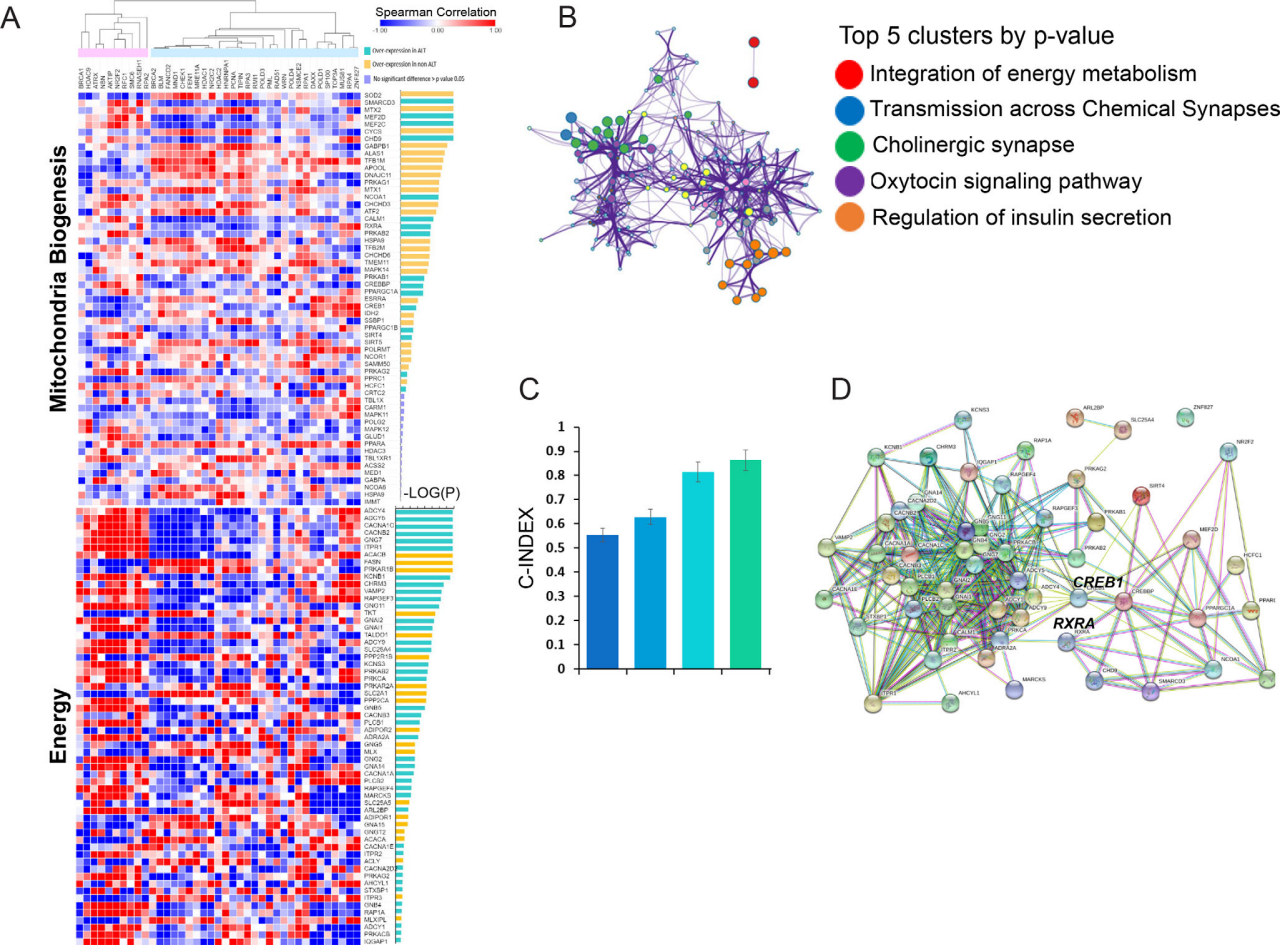
Mitochondrial biogenesis and energy metabolism genes are enriched in ALT. (A) Heatmap of Pearson correlations between mitochondrial biogenesis, energy metabolism, and ALT genes. *p*‐Values were calculated using the Wilcoxon test and were Benjamini–Hochberg‐adjusted. Correlations between the expression levels for ALT genes and mitochondrial biogenesis genes are shown. Statistical significance is shown as –log10[adjusted *p*], where *p* < 0.05 corresponds to –log10[adjusted *p*] > 1.3. Dark red and dark blue indicate highly significant negative and positive correlations, respectively. (B) Top five gene clusters of genes enriched in ALT. (C) Power of risk prediction (C‐index) with ALT signature, EMT signature, and energy signature as well as all signature genes for distinct ALT activity in GC. Dark blue: ALT‐like, Blue: EMT, Sky blue: Energy, Green: Energy+ALT‐like+EMT. (D) Protein‐protein interactions between mitochondrial biogenesis genes and energy metabolism genes (hub genes: *CREB1* and *RXRA*). Abbreviations: Alternative lengthening of telomeres (ALT); epithelial‐mesenchymal transition (EMT); gastric cancer (GC); and telomere maintenance mechanism (TMM)

To investigate drug repositioning for ALT‐like GC, we assessed candidate drug and target genes using the online tool GDSC.^10^ JW‐7‐52‐1, Sunitinib, VX‐680, and BI‐2536 were significantly enriched for high‐level ALT in GC (Figure 4A). Notably, we found that *NR3C1* (Figure 4B) was a hub gene among the candidate target genes for ALT‐like GC. *NR3C1* is regulated by RARA and NR2F2 (Figure 4C, Table S5). We further validated its clinical relevance using an independent GC dataset (TCGA STAD). Poor clinical outcomes were associated with upregulated *NR3C1* expression (Figure 4D). Moreover, the *NR3C1* level was significantly increased in our high‐ALT cohort (Figure 4E).

**FIGURE 4:**
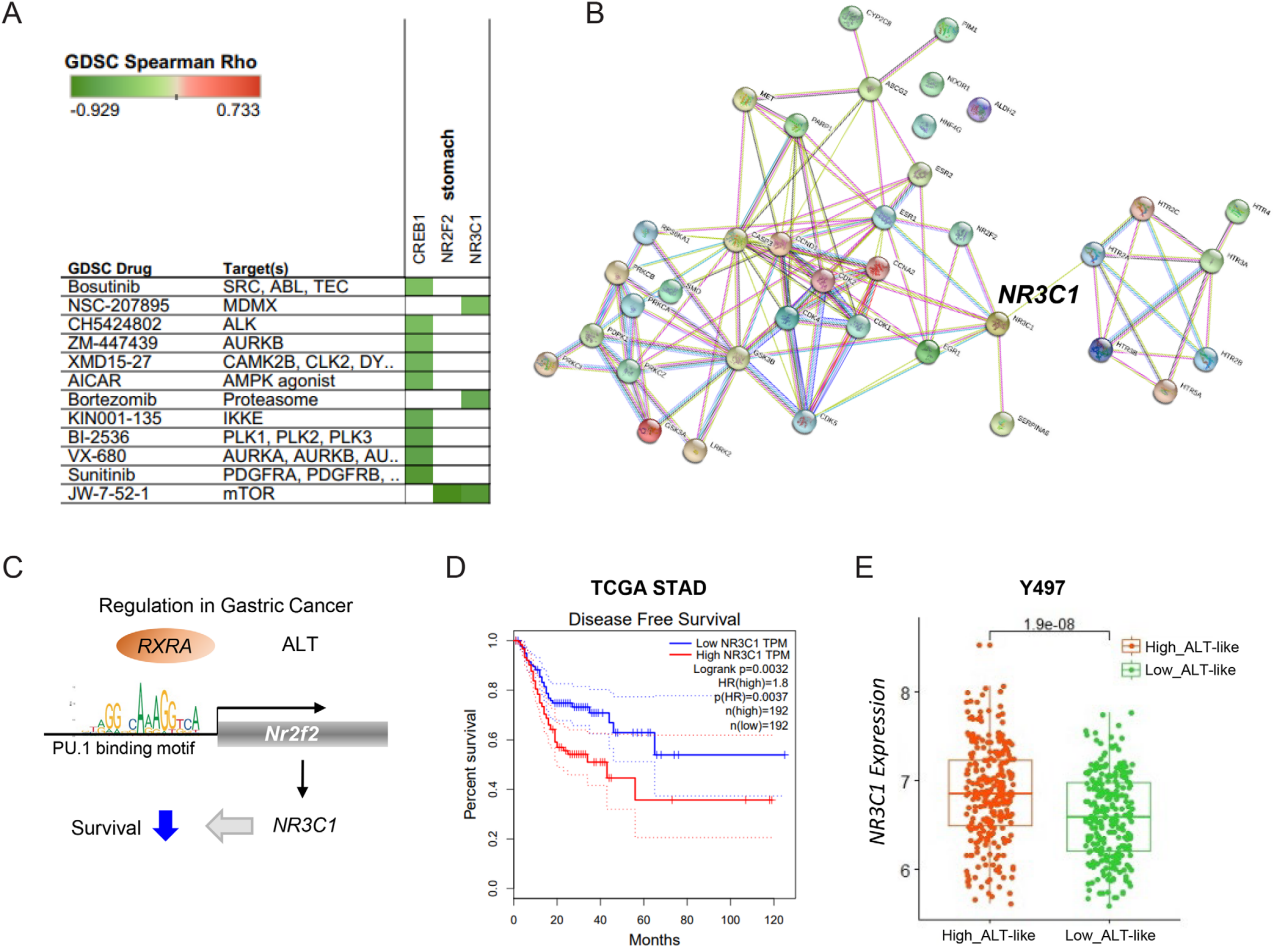
Predicted drug resistance and target genes in GC. (A) Prediction of drug and drug target genes using GDSC. (B) Protein‐protein interactions among predicted candidate genes related with ALT‐like in GC (hub gene: NR3C1). (C) Schematic diagram of regulation in GC. (D) Kaplan–Meier plots showing overall survival rates for NR3C1‐high and ‐low groups. *p*‐Values were calculated using the log‐rank test. *p* = 0.0032 for *NR3C1* expression in TCGA STAD. (E) Boxplot of *NR3C1* expression in high‐ALT and low‐ALT‐like tumors in our cohort. Abbreviations: Alternative lengthening of telomeres (ALT); gastric cancer (GC); and telomere maintenance mechanism (TMM)

In this study, we demonstrate that ALT‐like GC is associated with EMT, energy metabolic reprogramming, and mitochondrial biosynthesis. Additionally, we identified novel therapeutic targets specific to ALT‐like tumors. Our study provides a new model of telomere extension and potential therapeutic strategies for the ALT‐associated aggressive subtype of GC.

## AUTHOR CONTRIBUTIONS

Conceptualization: Ji‐Yong Sung; Methodology: Ji‐Yong Sung and Jae‐Ho Cheong; Data analysis: Ji‐Yong Sung; Writing—original draft preparation: Ji‐Yong Sung; Writing, review, and editing: Ji‐Yong Sung and Jae‐Ho Cheong; Supervision: Jae‐Ho Cheong; Project administration: Jae‐Ho Cheong; Funding acquisition: Jae‐Ho Cheong. Ji‐Yong Sung conceived and designed the overall study. Ji‐Yong Sung and Jae‐Ho Cheong contributed to the development of the hypothesis and analysis schemes. Ji‐Yong Sung performed the data analyses. All authors contributed to the interpretation of the results. Ji‐Yong Sung wrote the manuscript. Jae‐Ho Cheong revised, edited, and reviewed the manuscript. All authors have read and agreed to the published version of the manuscript.

## CONFLICT OF INTEREST

The authors declare that they have no conflict of interest.

## FUNDING INFORMATION

This research was supported by a grant from the KHIDI, funded by the Ministry of Health and Welfare (HI14C1324) and from the NRF, funded by the Ministry of Science and ICT (NRF‐2018R1A5A2025079), Republic of Korea.

## Supporting information

Supporting information

TableS1

TableS2

TableS3

TableS4

TableS5
