# Supplementary material for: Alternative lengthening of telomeres is mechanistically linked to potential therapeutic vulnerability in the stem‐like subtype of gastric cancer
Source: Clin Transl Med. 2021 Sep 14;11(9):e561. doi: 10.1002/ctm2.561 (PMC8438564; doi:10.1002/ctm2.561)
Supplement: Supplementary file 1 — Supporting information [file CTM2-11-e561-s003.docx]

Supplementary Information

for

Sung et al., Alternative Lengthening of Telomere in Gastric Cancer

**Supplementary Figures**

**Figure S1.** Molecular subtype landscape for Gastric Cancer (A) Boxplot of seven metabolic reprogramming signature activity across each molecular subtype (B) Kaplan Meier survival analysis for five molecular subtype (C) Immune landscape (D) Spearman correlation of metabolic signature in 497 gastric cancer patients (E) Pearson correlation of molecular subtypes (F) Cancer Hallmark of five molecular subtypes.

**Figure S2.** Telomere Maintenance Mechanism (TMM) for molecular subtypes in Gastric cancer (A) Six TMM signatures for molecular subtypes (B) Kaplan Meier survival analysis for 497 samples (C) Radar chart of correlation between proliferation and TMM signature (D) Similarity matrix of TMM for Y497 samples

**Figure S3.** Comparison between Telomere Maintenance Mechanism (TMM) and MSI states and telomere lengthening in TCGA STAD cohort.

**Figure S4.** Hallmark of ALT in Gastric Cancer (A) Spider plot of significant difference for oncogenes and tumor suppressor genes between ALT and non ALT (B) Boxplot of hetero chromatin and PDGFRB (C) Heatmap of cancer hallmark between ALT and non ALT (D) Pearson correlation of metabolic signatures and telomere maintenance mechanism

**Supplementary Tables**

**Table S1.** List of genes involved in the telomere maintenance mechanism

**Table S2.** List of significant signature genes involved in the telomere maintenance mechanism across cancer types

**Table S3.** List of significant gene ontology (GO) terms enriched in the ALT group and non ALT group

**Table S4.** List of significant gene ontology (GO) terms enriched in HDAC8 targeted genes

**Table S5.** List of NR2F2 binding prediction


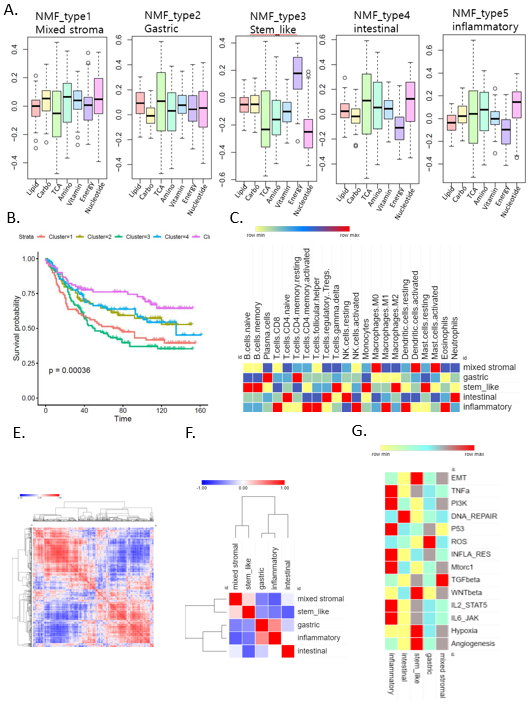


**Figure S1.** Molecular subtype landscape for Gastric Cancer (A) Boxplot of seven metabolic reprogramming signature activity across each molecular subtype (B) Kaplan Meier survival analysis for five molecular subtype (C) Immune landscape (D) Spearman correlation of metabolic signature in 497 gastric cancer patients (E) Pearson correlation of molecular subtypes (F) Cancer Hallmark of five molecular subtypes.


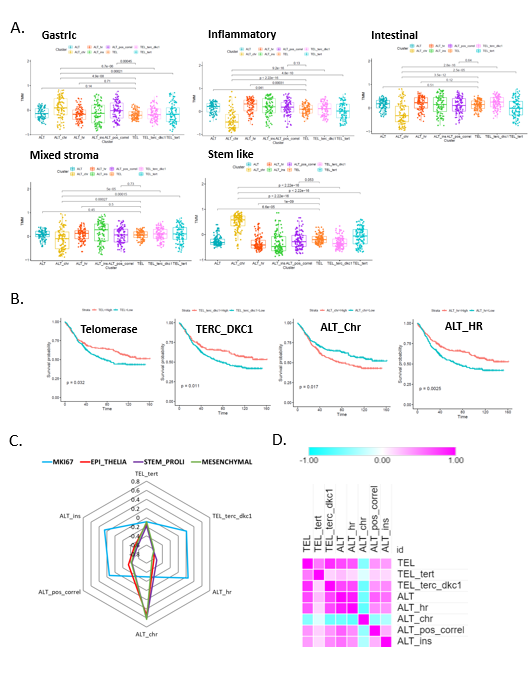


**Figure S2.** Telomere Maintenance Mechanism (TMM) for molecular subtypes in Gastric cancer (A) 6 TMM for molecular subtypes (B) Kaplan Meier survival analysis for 497 samples (C) Radar chart of correlation between proliferation and TMM signature (D) Similarity matrix of TMM for Y497 samples


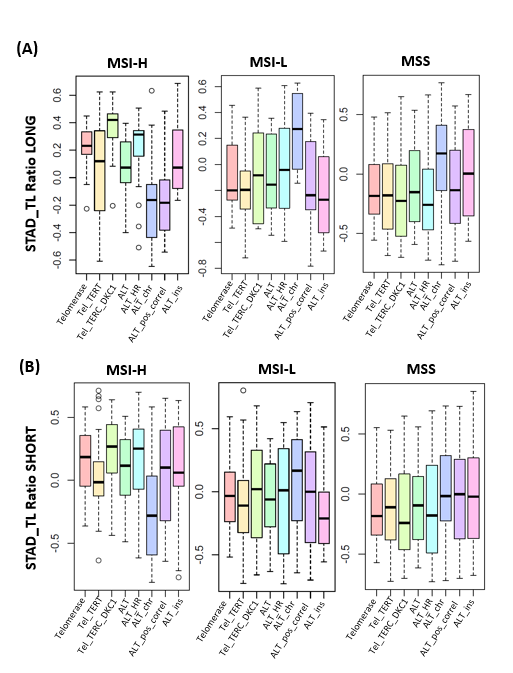


**Figure S3.** Comparison between Telomere Maintenance Mechanism (TMM) and MSI states and telomere lengthening in TCGA STAD cohort.


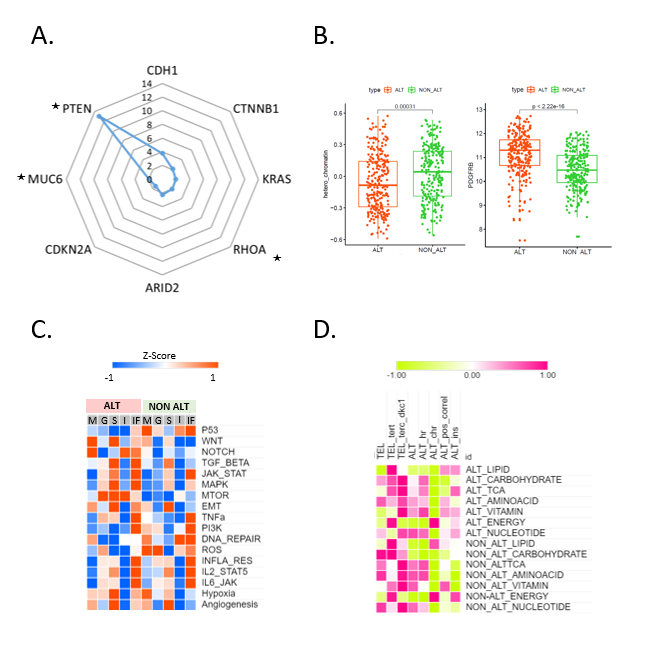


**Figure S4.** Hallmark of ALT in Gastric Cancer (A) Spider plot of significant difference for oncogenes and tumor suppressor genes between ALT and non ALT (B) Boxplot of hetero chromatin and PDGFRB (C) Heatmap of cancer hallmark between ALT and non ALT (D) Pearson correlation of metabolic signatures and telomere maintenance mechanism
