# Supplementary material for: Alternative lengthening of telomeres is mechanistically linked to potential therapeutic vulnerability in the stem‐like subtype of gastric cancer
Source: Clin Transl Med. 2021 Sep 14;11(9):e561. doi: 10.1002/ctm2.561 (PMC8438564; doi:10.1002/ctm2.561)
Supplement: Supplementary file 2 — TableS1 [file CTM2-11-e561-s005.pdf]

Table S1. List of genes involved in the telomere maintenance mechanism

TMM type	Pathway	Gene name	Description
Alternative Lengthening Telomere	Homologous Recombination pathway	*WRN*	WRN RecQ like helicase
Alternative Lengthening Telomere	Homologous Recombination pathway	*AKTIP*	AKT Interacting Protein
Alternative Lengthening Telomere	Homologous Recombination pathway	*POLD4*	DNA Polymerase Delta 4, Accessory Subunit
Alternative Lengthening Telomere	Homologous Recombination pathway	*POLD3*	DNA Polymerase Delta 3, Accessory Subunit
Alternative Lengthening Telomere	Homologous Recombination pathway	*POLD1*	DNA Polymerase Delta 1, Catalytic Subunit
Alternative Lengthening Telomere	Homologous Recombination pathway	*CBX5*	Chromobox 5
Alternative Lengthening Telomere	Homologous Recombination pathway	*HIRA*	Histone Cell Cycle Regulator
Alternative Lengthening Telomere	Homologous Recombination pathway	*FANCD2*	FA Complementation Group D2
Alternative Lengthening Telomere	Homologous Recombination pathway	*TIMELESS*	Timeless Circadian Regulator
Alternative Lengthening Telomere	Homologous Recombination pathway	*TIPIN*	TIMELESS Interacting Protein
Alternative Lengthening Telomere	Homologous Recombination pathway	*SMARCAL1*	SWI/SNF Related, Matrix Associated, Actin Dependent Regulator Of Chromatin, Subfamily A Like 1
Alternative Lengthening Telomere	Homologous Recombination pathway	*PCNA*	Proliferating Cell Nuclear Antigen
Alternative Lengthening Telomere	Homologous Recombination pathway	*CHEK1*	Checkpoint Kinase 1
Alternative Lengthening Telomere	Homologous Recombination pathway	*RNASEH1*	Ribonuclease H1
Alternative Lengthening Telomere	Homologous Recombination pathway	*MND1*	Meiotic Nuclear Divisions 1
Alternative Lengthening Telomere	Homologous Recombination pathway	*RAD51*	RAD51 Recombinase
Alternative Lengthening Telomere	Homologous Recombination pathway	*ATR*	ATR Serine/Threonine Kinase
Alternative Lengthening Telomere	Homologous Recombination pathway	*RAD52*	RAD52 Homolog, DNA Repair Protein
Alternative Lengthening Telomere	Homologous Recombination pathway	*SLX1A*	SLX1 Homolog A, Structure-Specific Endonuclease Subunit
Alternative Lengthening Telomere	Homologous Recombination pathway	*SLX1B*	SLX1 Homolog B, Structure-Specific Endonuclease Subunit
Alternative Lengthening Telomere	Homologous Recombination pathway	*SLX4*	SLX4 Structure-Specific Endonuclease Subunit
Alternative Lengthening Telomere	Homologous Recombination pathway	*BLM*	BLM RecQ like helicase
Alternative Lengthening Telomere	Homologous Recombination pathway	*RMI1*	RecQ Mediated Genome Instability 1
Alternative Lengthening Telomere	Homologous Recombination pathway	*RMI2*	RecQ Mediated Genome Instability 2
Alternative Lengthening Telomere	Homologous Recombination pathway	*PSMC3IP*	PSMC3 Interacting Protein
Alternative Lengthening Telomere	Homologous Recombination pathway	*TOP3A*	DNA Topoisomerase III Alpha
Alternative Lengthening Telomere	Homologous Recombination pathway	*POT1*	protection of telomeres 1
Alternative Lengthening Telomere	Homologous Recombination pathway	*RPA4*	Replication Protein A4
Alternative Lengthening Telomere	Homologous Recombination pathway	*RPA3*	Replication Protein A3
Alternative Lengthening Telomere	Homologous Recombination pathway	*HNRNPA1*	Heterogeneous Nuclear Ribonucleoprotein A1
Alternative Lengthening Telomere	Homologous Recombination pathway	*RPA2*	Replication Protein A2
Alternative Lengthening Telomere	Homologous Recombination pathway	*FEN1*	Flap Structure-Specific Endonuclease 1
Alternative Lengthening Telomere	Homologous Recombination pathway	*RPA1*	Replication Protein A1
Alternative Lengthening Telomere	Homologous Recombination pathway	*RFC1*	Replication Factor C Subunit 1
Alternative Lengthening Telomere	Homologous Recombination pathway	*BRCA2*	BRCA2 DNA Repair Associated

Alternative Lengthening Telomere	Chromatin Decompaction pathway	*NR2C2*	Nuclear Receptor Subfamily 2 Group C Member 2
Alternative Lengthening Telomere	Chromatin Decompaction pathway	*NR2F2*	nuclear receptor subfamily 2 group F member 2
Alternative Lengthening Telomere	Chromatin Decompaction pathway	*SUV39H2*	Suppressor Of Variegation 3-9 Homolog 2
Alternative Lengthening Telomere	Chromatin Decompaction pathway	*SUV39H1*	Suppressor Of Variegation 3-9 Homolog 1
Alternative Lengthening Telomere	Chromatin Decompaction pathway	*HDAC1*	histone deacetylase 1
Alternative Lengthening Telomere	Chromatin Decompaction pathway	*HDAC2*	histone deacetylase 2
Alternative Lengthening Telomere	Chromatin Decompaction pathway	*ZNF827*	Zinc Finger Protein 827
Alternative Lengthening Telomere	Chromatin Decompaction pathway	*KMT5C*	Lysine Methyltransferase 5C
Alternative Lengthening Telomere	Chromatin Decompaction pathway	*KMT5B*	Lysine Methyltransferase 5B
Alternative Lengthening Telomere	Chromatin Decompaction pathway	*HDAC9*	Histone Deacetylase 9
Alternative Lengthening Telomere	PML pathway	*SP100*	SP100 Nuclear Antigen
Alternative Lengthening Telomere	PML pathway	*BRCA1*	BRCA1 DNA Repair Associated
Alternative Lengthening Telomere	PML pathway	*PML*	Promyelocytic Leukemia
Alternative Lengthening Telomere	PML pathway	*GEN1*	GEN1 Holliday Junction 5' Flap Endonuclease
Alternative Lengthening Telomere	PML pathway	*MUS81*	MUS81 Structure-Specific Endonuclease Subunit
Alternative Lengthening Telomere	PML pathway	*EME1*	Essential Meiotic Structure-Specific Endonuclease 1
Alternative Lengthening Telomere	PML pathway	*NBN*	nibrin
Alternative Lengthening Telomere	PML pathway	*MRE11A*	MRE11 homolog, double strand break repair nuclease
Alternative Lengthening Telomere	PML pathway	*RAD50*	RAD50 double strand break repair protein
Alternative Lengthening Telomere	PML pathway	*SMC5*	Structural Maintenance Of Chromosomes 5
Alternative Lengthening Telomere	PML pathway	*SMC6*	Structural Maintenance Of Chromosomes 6
Alternative Lengthening Telomere	PML pathway	*NSMCE2*	NSE2 (MMS21) Homolog, SMC5-SMC6 Complex SUMO Ligase
Alternative Lengthening Telomere	Telomere Instability	*ATRX*	ATRX Chromatin Remodeler
Alternative Lengthening Telomere	Telomere Instability	*DAXX*	Death Domain Associated Protein
Alternative Lengthening Telomere	Telomere Instability	*FEN1*	Flap Structure-Specific Endonuclease 1
Alternative Lengthening Telomere	Telomere Instability	*TERT*	telomerase reverse transcriptase
Telomerase	TERT pathway	*KPNA1*	Karyopherin Subunit Alpha 1
Telomerase	TERT pathway	*KPNB1*	Karyopherin Subunit Beta 1
Telomerase	TERT pathway	*IPO7*	Importin 7
Telomerase	TERT pathway	*HSPA1A*	Heat Shock Protein Family A (Hsp70) Member 1A
Telomerase	TERT pathway	*PTGES3*	Prostaglandin E Synthase 3
Telomerase	TERT pathway	*RANBP2*	RAN Binding Protein 2
Telomerase	TERT pathway	*XRN1*	5'-3' Exoribonuclease 1
Telomerase	TERT pathway	*DCP2*	Decapping MRNA 2
Telomerase	TERT pathway	*EXOSC3*	Exosome Component 3
Telomerase	TERT pathway	*DIS3*	DIS3 Homolog, Exosome Endoribonuclease And 3'-5' Exoribonuclease
Telomerase	TERT pathway	*PKC*	Protein Kinase C Alpha

Telomerase	TERT pathway	*ACD*	ACD shelterin complex subunit and telomerase recruitment factor
Telomerase	TERT pathway	*PINX1*	PIN2 (TERF1) Interacting Telomerase Inhibitor 1
Telomerase	TERT pathway	*TNKS1BP1*	Tankyrase 1 Binding Protein 1
Telomerase	TERT pathway	*TERF2IP*	TERF2 interacting protein
Telomerase	TERT pathway	*WRAP53*	WD Repeat Containing Antisense To TP53
Telomerase	TERT pathway	*HSP90AA1*	heat shock protein 90 alpha family class A member 1
Telomerase	TERT pathway	*HSP90AA2*	Heat Shock Protein 90 Alpha Family Class A Member 2, Pseudogene
Telomerase	TERT pathway	*HSP90AB1*	Heat Shock Protein 90 Alpha Family Class B Member 1
Telomerase	TERT pathway	*ATM*	ATM Serine/Threonine Kinase
Telomerase	TERT pathway	*HSP90*	Heat Shock Protein 90 Alpha Family Class A Member 1
Telomerase	TERT pathway	*ABL1*	ABL Proto-Oncogene 1, Non-Receptor Tyrosine Kinase
Telomerase	TERT pathway	*STUB1*	STIP1 Homology And U-Box Containing Protein 1
Telomerase	TERT pathway	*TERT*	telomerase reverse transcriptase
Telomerase	TERC_DKC1 pathway	*SRRT*	Serrate, RNA Effector Molecule
Telomerase	TERC_DKC1 pathway	*NCBP2*	Nuclear Cap Binding Protein Subunit 2
Telomerase	TERC_DKC1 pathway	*NCBP1*	Nuclear Cap Binding Protein Subunit 1
Telomerase	TERC_DKC1 pathway	*EXOSC10*	Exosome Component 10
Telomerase	TERC_DKC2 pathway	*ZCCHC7*	Zinc Finger CCHC-Type Containing 7
Telomerase	TERC_DKC1 pathway	*ZCCHC8*	Zinc Finger CCHC-Type Containing 8
Telomerase	TERC_DKC1 pathway	*MTR4*	Mtr4 Exosome RNA Helicase
Telomerase	TERC_DKC1 pathway	*PAPD5*	Terminal Nucleotidyltransferase 4B
Telomerase	TERC_DKC1 pathway	*PABPN1*	Poly(A) Binding Protein Nuclear 1
Telomerase	TERC_DKC1 pathway	*PARN*	Poly(A)-Specific Ribonuclease
Telomerase	TERC_DKC1 pathway	*FXR1*	FMR1 Autosomal Homolog 1
Telomerase	TERC_DKC1 pathway	*NAF1*	Nuclear Assembly Factor 1 Ribonucleoprotein
Telomerase	TERC_DKC1 pathway	*GAR1*	GAR1 Ribonucleoprotein
Telomerase	TERC_DKC1 pathway	*NOP10*	NOP10 Ribonucleoprotein
Telomerase	TERC_DKC1 pathway	*NHP2*	NHP2 Ribonucleoprotein
Telomerase	TERC_DKC1 pathway	*RUVBL1*	RuvB Like AAA ATPase 1
Telomerase	TERC_DKC1 pathway	*RUVBL2*	RuvB Like AAA ATPase 2
Telomerase	TERC_DKC2 pathway	*TERC*	Telomerase RNA Component
Telomerase	TERC_DKC3 pathway	*DKC1*	dyskerin pseudouridine synthase 1
