# Supplementary material for: Alternative lengthening of telomeres is mechanistically linked to potential therapeutic vulnerability in the stem‐like subtype of gastric cancer
Source: Clin Transl Med. 2021 Sep 14;11(9):e561. doi: 10.1002/ctm2.561 (PMC8438564; doi:10.1002/ctm2.561)
Supplement: Supplementary file 3 — TableS2 [file CTM2-11-e561-s002.pdf]

Table S2. List of significant signature genes involved in the telomere maintenance mechanism across cancer types

Chromatin Decompaction Type	TMM type	Gene name	P-value
High	Alternative Lengthening Telomere	*AKTIP*	0.075
High	Alternative Lengthening Telomere	*ATRX*	0.01241
High	Alternative Lengthening Telomere	*BLM*	2.751E-15
High	Alternative Lengthening Telomere	*BRCA1*	0.4623
High	Alternative Lengthening Telomere	*BRCA2*	5.497E-10
High	Alternative Lengthening Telomere	*CHEK1*	2.2E-16
High	Alternative Lengthening Telomere	*DAXX*	0.003197
High	Alternative Lengthening Telomere	*FANCD2*	2.2E-16
High	Alternative Lengthening Telomere	*FEN1*	2.2E-16
High	Alternative Lengthening Telomere	*HDAC1*	1.294E-13
High	Alternative Lengthening Telomere	*HDAC2*	0.01494
High	Alternative Lengthening Telomere	*HDAC9*	0.4194
High	Alternative Lengthening Telomere	*HNRNPA1*	2.2E-16
High	Alternative Lengthening Telomere	*MND1*	2.2E-16
High	Alternative Lengthening Telomere	*MRE11A*	0.00000075
High	Alternative Lengthening Telomere	*MUS81*	0.05608
High	Alternative Lengthening Telomere	*NBN*	7.098E-10
High	Alternative Lengthening Telomere	*NR2C2*	1.71E-13
High	Alternative Lengthening Telomere	*NR2F2*	2.2E-16
High	Alternative Lengthening Telomere	*NSMCE2*	5.935E-08
High	Alternative Lengthening Telomere	*PCNA*	2.2E-16
High	Alternative Lengthening Telomere	*PML*	0.00002989
High	Alternative Lengthening Telomere	*POLD1*	9.421E-12
High	Alternative Lengthening Telomere	*POLD3*	2.776E-06
High	Alternative Lengthening Telomere	*POLD4*	0.2556
High	Alternative Lengthening Telomere	*RAD51*	2.2E-16

High	Alternative Lengthening Telomere	*RFC1*	0.07714
High	Alternative Lengthening Telomere	*RMI1*	1.309E-11
High	Alternative Lengthening Telomere	*RNASEH1*	0.02288
High	Alternative Lengthening Telomere	*RPA1*	2.311E-11
High	Alternative Lengthening Telomere	*RPA2*	0.02212
High	Alternative Lengthening Telomere	*RPA3*	2.614E-14
High	Alternative Lengthening Telomere	*RPA4*	0.001006
High	Alternative Lengthening Telomere	*SMC6*	0.005371
High	Alternative Lengthening Telomere	*SP100*	0.002988
High	Alternative Lengthening Telomere	*TIPIN*	2.2E-16
High	Alternative Lengthening Telomere	*TOP3A*	1.792E-07
High	Alternative Lengthening Telomere	*WRN*	0.004538
High	Alternative Lengthening Telomere	*ZNF827*	2.2E-16
Low	Telomerase	*DKC1*	2.2E-16
Low	Telomerase	*EXOSC3*	2.2E-16
Low	Telomerase	*NCBP2*	2.2E-16
Low	Telomerase	*RUVBL1*	2.2E-16
Low	Telomerase	*RUVBL2*	2.2E-16
Low	Telomerase	*NOP10*	7.826E-15
Low	Telomerase	*GAR1*	1.018E-12
Low	Telomerase	*NCBP1*	2.823E-08
Low	Telomerase	*NHP2*	1.735E-07
Low	Telomerase	*HSP90AB1*	2.009E-07
Low	Telomerase	*PTGES3*	2.742E-07
Low	Telomerase	*KPNA1*	4.551E-07
Low	Telomerase	*ABL1*	9.298E-07
Low	Telomerase	*ZCCHC8*	6.154E-06
Low	Telomerase	*TERC*	7.012E-06

Low	Telomerase	*FXR1*	0.00001383
Low	Telomerase	*ZCCHC7*	0.00005004
Low	Telomerase	*IPO7*	0.0000544
Low	Telomerase	*KPNB1*	0.0001605
Low	Telomerase	*HSP90AA1*	0.0001931
Low	Telomerase	*WRAP53*	0.0003903
Low	Telomerase	*SRRT*	0.002331
Low	Telomerase	*RANBP2*	0.01758
Low	Telomerase	*HSPA1A*	0.04167
Low	Telomerase	*DCP2*	0.1433
Low	Telomerase	*STUB1*	0.2036
Low	Telomerase	*XRN1*	0.27
Low	Telomerase	*PAPD5*	0.3913
Low	Telomerase	*PARN*	0.3961
Low	Telomerase	*DIS3*	0.6799
Low	Telomerase	*EXOSC10*	0.7589
