# Supplementary material for: Alternative lengthening of telomeres is mechanistically linked to potential therapeutic vulnerability in the stem‐like subtype of gastric cancer
Source: Clin Transl Med. 2021 Sep 14;11(9):e561. doi: 10.1002/ctm2.561 (PMC8438564; doi:10.1002/ctm2.561)
Supplement: Supplementary file 4 — TableS3 [file CTM2-11-e561-s006.pdf]

**Table S3.** List of significant gene ontology (GO) terms enriched in the ALT-like group and non ALT group

| Chromatin Decompaction Type | Prognosis | Category | Term | Description | LogP | Log(q-value) |
|---|---|---|---|---|---|---|
| High | Poor | GO Biological Processes | GO:0006397 | mRNA processing | -10.589 | -6.269871994 |
| High | Poor | GO Biological Processes | GO:0016570 | histone modification | -9.05492 | -5.250122606 |
| High | Poor | GO Biological Processes | GO:1902275 | regulation of chromatin organization | -8.98277 | -5.250122606 |
| High | Poor | GO Biological Processes | GO:0044257 | cellular protein catabolic process | -8.5671 | -4.951781437 |
| High | Poor | GO Biological Processes | GO:0006914 | autophagy | -8.42579 | -4.951781437 |
| High | Poor | Reactome Gene Sets | R-HSA-3247509 | Chromatin modifying enzymes | -7.62659 | -4.483576345 |
| High | Poor | GO Biological Processes | GO:0007017 | microtubule-based process | -7.05963 | -3.944639698 |
| High | Poor | Reactome Gene Sets | R-HSA-983168 | Antigen processing: Ubiquitination & F | -6.47015 | -3.429800576 |
| High | Poor | GO Biological Processes | GO:0071824 | protein-DNA complex subunit organiza | -6.09352 | -3.192262995 |
| High | Poor | Reactome Gene Sets | R-HSA-69278 | Cell Cycle, Mitotic | -6.0812 | -3.192262995 |
| High | Poor | GO Biological Processes | GO:0050657 | nucleic acid transport | -5.83526 | -3.022137766 |
| High | Poor | GO Biological Processes | GO:0051640 | organelle localization | -5.82829 | -3.022137766 |
| High | Poor | GO Biological Processes | GO:0010564 | regulation of cell cycle process | -5.80317 | -3.01554104 |
| High | Poor | GO Biological Processes | GO:0022411 | cellular component disassembly | -5.65267 | -2.924632038 |
| High | Poor | Reactome Gene Sets | R-HSA-9006925 | Intracellular signaling by second mess | -5.57385 | -2.871581804 |
| High | Poor | KEGG Pathway | hsa01522 | Endocrine resistance | -5.31032 | -2.672456375 |
| High | Poor | Reactome Gene Sets | R-HSA-5696399 | Global Genome Nucleotide Excision R | -5.26521 | -2.636301805 |
| High | Poor | GO Biological Processes | GO:0010256 | endomembrane system organization | -5.14237 | -2.547540214 |
| High | Poor | GO Biological Processes | GO:0007034 | vacuolar transport | -5.07027 | -2.502829784 |
| Low | Good | GO Biological Processes | GO:0031349 | positive regulation of defense respons | -11.3638 | -7.04471182 |
| Low | Good | GO Biological Processes | GO:0034123 | positive regulation of toll-like receptor | -9.75064 | -5.908659302 |
| Low | Good | GO Biological Processes | GO:0002274 | myeloid leukocyte activation | -9.05642 | -5.515468777 |
| Low | Good | GO Biological Processes | GO:0001819 | positive regulation of cytokine product | -8.1197 | -4.72885965 |
| Low | Good | GO Biological Processes | GO:0002697 | regulation of immune effector process | -7.55319 | -4.322781154 |
| Low | Good | GO Biological Processes | GO:0042110 | T cell activation | -7.4357 | -4.262718944 |
| Low | Good | Reactome Gene Sets | R-HSA-1280218 | Adaptive Immune System | -6.88873 | -3.848382261 |
| Low | Good | GO Biological Processes | GO:0034154 | toll-like receptor 7 signaling pathway | -6.37842 | -3.381533764 |
| Low | Good | GO Biological Processes | GO:0032647 | regulation of interferon-alpha producti | -6.09954 | -3.227589884 |
| Low | Good | GO Biological Processes | GO:0032103 | positive regulation of response to exte | -5.71845 | -2.955645623 |
| Low | Good | GO Biological Processes | GO:0019221 | cytokine-mediated signaling pathway | -5.52913 | -2.812084855 |
| Low | Good | GO Biological Processes | GO:0051345 | positive regulation of hydrolase activity | -5.32993 | -2.70979488 |
| Low | Good | GO Biological Processes | GO:0009617 | response to bacterium | -5.04979 | -2.494112201 |

| | | | | | | |
|---|---|---|---|---|---|---|
| Low | Good | GO Biological Processes | GO:0002831 | regulation of response to biotic stimulu | -5.00653 | -2.465574516 |
| Low | Good | GO Biological Processes | GO:0032760 | positive regulation of tumor necrosis fa | -4.54908 | -2.146005969 |
| Low | Good | GO Biological Processes | GO:0001909 | leukocyte mediated cytotoxicity | -4.54083 | -2.146005969 |
| Low | Good | GO Biological Processes | GO:0033632 | regulation of cell-cell adhesion mediat | -4.52092 | -2.136316396 |
| Low | Good | GO Biological Processes | GO:0072507 | divalent inorganic cation homeostasis | -4.51208 | -2.134368006 |
| Low | Good | GO Biological Processes | GO:0034341 | response to interferon-gamma | -4.11805 | -1.836372035 |
| Low | Good | GO Biological Processes | GO:0001821 | histamine secretion | -4.05752 | -1.7802587 |
