# Supplementary material for: Alternative lengthening of telomeres is mechanistically linked to potential therapeutic vulnerability in the stem‐like subtype of gastric cancer
Source: Clin Transl Med. 2021 Sep 14;11(9):e561. doi: 10.1002/ctm2.561 (PMC8438564; doi:10.1002/ctm2.561)
Supplement: Supplementary file 5 — TableS4 [file CTM2-11-e561-s001.pdf]

Table S4. List of significant gene ontology (GO) terms enriched in HDAC8 targeted genes

TFs	Category	Term	Description	LogP	Log(q-value)
HDAC8	Canonical Pathways	M8	PID ENDOTHELIN PATHWAY	-5.510813012	-1.421305
HDAC8	Reactome Gene Sets	R-HSA-9029558	NR1H2 & NR1H3 regulate gene expression linked to lipogenesis	-5.439381108	-1.421305
HDAC8	GO Biological Processes	GO:0031663	lipopolysaccharide-mediated signaling pathway	-4.226300029	-0.7747167
HDAC8	Canonical Pathways	M11	PID PRL SIGNALING EVENTS PATHWAY	-4.131156321	-0.7662928
HDAC8	GO Biological Processes	GO:0060761	negative regulation of response to cytokine stimulus	-3.731195947	-0.7172954
HDAC8	KEGG Pathway	hsa04932	Non-alcoholic fatty liver disease (NAFLD)	-3.704790945	-0.7172954
HDAC8	KEGG Pathway	hsa04540	Gap junction	-3.550899947	-0.6982558
HDAC8	Reactome Gene Sets	R-HSA-109582	Hemostasis	-3.421095587	-0.6756161
HDAC8	GO Biological Processes	GO:0010821	regulation of mitochondrion organization	-3.220152609	-0.5542591
HDAC8	GO Biological Processes	GO:0051817	modification of morphology or physiology of other organism involved in symbiotic interaction	-3.082662355	-0.4886903
HDAC8	Canonical Pathways	M220	PID CASPASE PATHWAY	-3.067433627	-0.4886903
HDAC8	Reactome Gene Sets	R-HSA-429914	Deadenylation-dependent mRNA decay	-2.996293999	-0.4553392
HDAC8	GO Biological Processes	GO:0010256	endomembrane system organization	-2.92931162	-0.416257
HDAC8	GO Biological Processes	GO:0048598	embryonic morphogenesis	-2.863795513	-0.3771984
HDAC8	GO Biological Processes	GO:0042692	muscle cell differentiation	-2.572009426	-0.3136012
HDAC8	GO Biological Processes	GO:0008015	blood circulation	-2.476648228	-0.2613459
HDAC8	GO Biological Processes	GO:1902532	negative regulation of intracellular signal transduction	-2.361991273	-0.2258524
HDAC8	GO Biological Processes	GO:0071897	DNA biosynthetic process	-2.231394021	-0.1864458
HDAC8	GO Biological Processes	GO:0071156	regulation of cell cycle arrest	-2.186503394	-0.1629787
HDAC8	GO Biological Processes	GO:0051648	vesicle localization	-2.174588724	-0.1629787
