# Supplementary material for: Alternative lengthening of telomeres is mechanistically linked to potential therapeutic vulnerability in the stem‐like subtype of gastric cancer
Source: Clin Transl Med. 2021 Sep 14;11(9):e561. doi: 10.1002/ctm2.561 (PMC8438564; doi:10.1002/ctm2.561)
Supplement: Supplementary file 6 — TableS5 [file CTM2-11-e561-s004.pdf]

Table S5. List of NR2F2 binding prediction

TF	JASPAR ID	Class	Family	Tax Group	IC	GC Content	Target gene hits	Target gene non-hits	Background gene hits	Background gene non-hits	Target TFBS hits	Target TFBS nucleotide rate	Background TFBS hits	Background TFBS nucleotide rate	Z-score	Fisher score
RREB1	MA0073.1	Zinc-coordinating	BetaBetaAlpha-zinc finger	vertebrates	22.278	0.623	1	0	1520	23232	4	0.00622	1839	0.00102	18.307	2.79
PPARG::RXRA	MA0065.2	Zinc-coordinating	Hormone-nuclear Receptor	vertebrates	11.663	0.547	1	0	6067	18685	10	0.0117	11080	0.00462	11.714	1.406
MZF1_5-13	MA0057.1	Zinc-coordinating	BetaBetaAlpha-zinc finger	vertebrates	9.4	0.588	1	0	13425	11327	47	0.0365	77649	0.0216	11.644	0.612
MZF1_1-4	MA0056.1	Zinc-coordinating	BetaBetaAlpha-zinc finger	vertebrates	8.586	0.725	1	0	16863	7889	107	0.0499	201000	0.0335	10.304	0.384
SPIB	MA0081.1	Winged	Helix-Turn-Helix Ets	vertebrates	9.06	0.466	1	0	17547	7205	92	0.0501	175639	0.0342	9.899	0.344
PLAG1	MA0163.1	Zinc-coordinating	BetaBetaAlpha-zinc finger	vertebrates	19.352	0.798	1	0	1971	22781	3	0.00326	2462	0.000958	8.316	2.53
RXRA::VDR	MA0074.1	Zinc-coordinating	Hormone-nuclear Receptor	vertebrates	20.451	0.527	1	0	453	24299	1	0.00117	475	0.000198	7.49	3.999
SPI1	MA0080.2	Winged	Helix-Turn-Helix Ets	vertebrates	9.64	0.435	1	0	15792	8960	49	0.0267	95792	0.0186	6.701	0.449
FOXO3	MA0157.1	Winged	Helix-Turn-Helix Forkhead	vertebrates	11.734	0.298	1	0	13611	11141	36	0.0224	69410	0.0154	6.365	0.598
FEV	MA0156.1	Winged	Helix-Turn-Helix Ets	vertebrates	12.121	0.442	1	0	15402	9350	41	0.0255	81722	0.0182	6.19	0.474
CTCF	MA0139.1	Zinc-coordinating	BetaBetaAlpha-zinc finger	vertebrates	17.205	0.645	1	0	3236	21516	3	0.00443	3982	0.0021	5.669	2.034
HNF4A	MA0114.1	Zinc-coordinating	Hormone-nuclear Receptor	vertebrates	9.617	0.522	1	0	6389	18363	7	0.00707	11240	0.00406	5.305	1.354
NF-kappaB	MA0061.1	Ig-fold	Rel vertebrate	13.345	0.621	1	0	8381	16371	11	0.00855	18865	0.00524	5.135	1.083	
CEBPA	MA0102.2	Zipper-Type	Leucine Zipper	vertebrates	8.712	0.358	1	0	12828	11924	27	0.0189	55606	0.0139	4.787	0.657
IRF2	MA0051.1	Winged	Helix-Turn-Helix IRF	vertebrates	21.134	0.412	1	0	844	23908	1	0.0014	923	0.000462	4.745	3.377
Ddit3::Cebpa	MA0019.1	Zipper-Type	Leucine Zipper	vertebrates	11.652	0.483	1	0	5576	19176	6	0.0056	9754	0.00325	4.592	1.49
SP1	MA0079.2	Zinc-coordinating	BetaBetaAlpha-zinc finger	vertebrates	11.129	0.82	1	0	12391	12361	36	0.028	79797	0.0222	4.444	0.692
ESR2	MA0258.1	Zinc-coordinating	Hormone-nuclear Receptor	vertebrates	13.618	0.546	1	0	2219	22533	2	0.0028	2670	0.00134	4.422	2.411
HNF1A	MA0046.1	Helix-Turn-Helix	Homeo vertebrate	15.548	0.259	1	0	3044	21708	3	0.00326	4352	0.00169	4.228	2.095	
NFATC2	MA0152.1	Ig-fold	Rel vertebrate	9.859	0.346	1	0	15176	9576	44	0.0239	97757	0.019	4.058	0.489	
Nobox	MA0125.1	Helix-Turn-Helix	Homeo vertebrate	9.573	0.26	1	0	13558	11194	35	0.0218	77190	0.0172	3.987	0.602	
NR4A2	MA0160.1	Zinc-coordinating	Hormone-nuclear Receptor	vertebrates	10.165	0.491	1	0	13896	10856	28	0.0174	60034	0.0133	3.98	0.577
TEAD1	MA0090.1	Helix-Turn-Helix	Homeo vertebrate	15.678	0.507	1	0	5222	19530	5	0.00466	8446	0.00282	3.871	1.556	
FOXD1	MA0031.1	Winged	Helix-Turn-Helix Forkhead	vertebrates	11.926	0.275	1	0	13087	11665	29	0.018	62921	0.014	3.869	0.637
Gata1	MA0035.2	Zinc-coordinating	GATA vertebrate	10.878	0.373	1	0	14350	10402	33	0.0282	75905	0.0232	3.748	0.545	
NFKB1	MA0105.1	Ig-fold	Rel vertebrate	15.627	0.758	1	0	3946	20806	4	0.00342	6372	0.00195	3.688	1.836	
INSM1	MA0155.1	Zinc-coordinating	BetaBetaAlpha-zinc finger	vertebrates	14.862	0.667	1	0	7588	17164	9	0.00839	17685	0.0059	3.642	1.182
SRF	MA0083.1	Other	Alpha-Helix MADS	vertebrates	17.965	0.466	1	0	908	23844	1	0.000933	1019	0.00034	3.41	3.304
Zfx	MA0146.1	Zinc-coordinating	BetaBetaAlpha-zinc finger	vertebrates	13.077	0.749	1	0	9872	14880	15	0.0163	33238	0.0129	3.367	0.919
Sox17	MA0078.1	Other	Alpha-Helix High	Mobility	Group	vertebrates	10.502	0.384	1	0	13879	10873	29	0.0203	65883	0.0165
TBP	MA0108.2	Beta-sheet	TATA-binding vertebrate	10.086	0.377	1	0	10389	14363	15	0.0175	33775	0.0141	3.246	0.868	
Nr2e3	MA0164.1	Zinc-coordinating	Hormone-nuclear Receptor	vertebrates	12.028	0.373	1	0	6354	18398	7	0.00381	12287	0.00239	3.205	1.36
IRF1	MA0050.1	Winged	Helix-Turn-Helix IRF	vertebrates	16.008	0.383	1	0	6075	18677	6	0.0056	11517	0.00384	3.149	1.405
ZNF354C	MA0130.1	Zinc-coordinating	BetaBetaAlpha-zinc finger	vertebrates	8.958	0.615	1	0	17566	7186	85	0.0396	207738	0.0346	3.081	0.343
Klf4	MA0039.2	Zinc-coordinating	BetaBetaAlpha-zinc finger	vertebrates	12.618	0.771	1	0	13645	11107	37	0.0288	88731	0.0247	2.972	0.596
Pax6	MA0069.1	Helix-Turn-Helix	Homeo vertebrate	13.798	0.432	1	0	1218	23534	1	0.00109	1377	0.000536	2.518	3.011	
Myb	MA0100.1	Helix-Turn-Helix	Myb vertebrate	9.883	0.591	1	0	13518	11234	25	0.0155	58600	0.013	2.48	0.605	
ZEB1	MA0103.1	Zinc-coordinating	BetaBetaAlpha-zinc finger	vertebrates	8.305	0.557	1	0	17454	7298	69	0.0322	171132	0.0285	2.458	0.349
REL	MA0101.1	Ig-fold	Rel vertebrate	10.515	0.559	1	0	10514	14238	13	0.0101	29250	0.00813	2.448	0.856	
RUNX1	MA0002.2	Ig-fold	Runt vertebrate	10.401	0.502	1	0	13641	11111	24	0.0205	58648	0.0179	2.184	0.596	
STAT1	MA0137.2	Ig-fold	Stat vertebrate	13.119	0.452	1	0	6394	18358	5	0.00583	10990	0.00458	2.032	1.353	
ELF5	MA0136.1	Winged	Helix-Turn-Helix Ets	vertebrates	8.693	0.429	1	0	16547	8205	46	0.0322	117435	0.0294	1.861	0.403
E2F1	MA0024.1	Winged	Helix-Turn-Helix E2F	vertebrates	13.838	0.625	1	0	8113	16639	8	0.00497	17630	0.00392	1.845	1.115
Prrx2	MA0075.1	Helix-Turn-Helix	Homeo vertebrate	9.063	0.027	1	0	15063	9689	48	0.0187	123270	0.0171	1.301	0.497	
YY1	MA0095.1	Zinc-coordinating	BetaBetaAlpha-zinc finger	vertebrates	8.101	0.51	1	0	17190	7562	63	0.0294	165054	0.0275	1.264	0.365
Arnt::Ahr	MA0006.1	Zipper-Type	Helix-Loop-Helix vertebrate	9.532	0.715	1	0	13597	11155	26	0.0121	66086	0.011	1.161	0.599	
FOXA1	MA0148.1	Winged	Helix-Turn-Helix Forkhead	vertebrates	12.533	0.332	1	0	13355	11397	24	0.0205	63112	0.0193	0.981	0.617
Zfp423	MA0116.1	Zinc-coordinating	BetaBetaAlpha-zinc finger	vertebrates	17.925	0.679	1	0	5691	19061	4	0.00466	10149	0.00423	0.689	1.47
Lhx3	MA0135.1	Helix-Turn-Helix	Homeo vertebrate	16.354	0.131	1	0	5806	18946	5	0.00505	12758	0.00461	0.678	1.45	
Esrrb	MA0141.1	Zinc-coordinating	Hormone-nuclear Receptor	vertebrates	12.806	0.524	1	0	10167	14585	10	0.00933	26346	0.00878	0.612	0.89
NR2F1	MA0017.1	Zinc-coordinating	Hormone-nuclear Receptor	vertebrates	15.924	0.478	1	0	3735	21017	2	0.00218	5073	0.00197	0.419	1.891
Foxa2	MA0047.2	Winged	Helix-Turn-Helix Forkhead	vertebrates	13.268	0.315	1	0	11656	13096	16	0.0149	43535	0.0145	0.349	0.753
Evi1	MA0029.1	Zinc-coordinating	BetaBetaAlpha-zinc finger	vertebrates	17.909	0.28	1	0	1931	22821	1	0.00109	2457	0.000956	0.343	2.55
ELK1	MA0028.1	Winged	Helix-Turn-Helix Ets	vertebrates	8.812	0.568	1	0	13642	11110	20	0.0155	54748	0.0152	0.272	0.596
Gfi	MA0038.1	Zinc-coordinating	BetaBetaAlpha-zinc finger	vertebrates	9.47	0.391	1	0	13731	11021	24	0.0187	65844	0.0183	0.27	0.589
RORA_1	MA0071.1	Zinc-coordinating	Hormone-nuclear Receptor	vertebrates	13.19	0.424	1	0	8624	16128	7	0.00544	18895	0.00525	0.238	1.054
MIZF	MA0131.1	Zinc-coordinating	BetaBetaAlpha-zinc finger	vertebrates	13.197	0.61	1	0	2080	22672	1	0.000777	2497	0.000694	0.192	2.476
AP1	MA0099.2	Zipper-Type	Leucine Zipper	vertebrates	9.193	0.405	1	0	16390	8362	41	0.0223	113253	0.022	0.185	0.412
Pdx1	MA0132.1	Helix-Turn-Helix	Homeo vertebrate	9.04	0.194	1	0	15507	9245	51	0.0238	140973	0.0235	0.181	0.468	
HOXA5	MA0158.1	Helix-Turn-Helix	Homeo vertebrate	8.759	0.315	1	0	17629	7123	74	0.046	205993	0.0458	0.099	0.339	
Sox5	MA0087.1	Other	Alpha-Helix High	Mobility	Group	vertebrates	10.831	0.199	1	0	13087	11665	26	0.0141	72042	0.014
RELA	MA0107.1	Ig-fold	Rel vertebrate	14.757	0.567	1	0	7171	17581	5	0.00389	13690	0.0038	0.08	1.239	
Foxq1	MA0040.1	Winged	Helix-Turn-Helix Forkhead	vertebrates	14.07	0.202	1	0	8007	16745	8	0.00684	22088	0.00675	0.069	1.129
Nkx2-5	MA0063.1	Helix-Turn-Helix	Homeo vertebrate	8.27	0.218	1	0	16973	7779	70	0.0381	197210	0.0384	-0.185	0.377	
SOX9	MA0077.1	Other	Alpha-Helix High	Mobility	Group	vertebrates	9.079	0.358	1	0	11417	13335	14	0.00979	41403	0.0104
Spz1	MA0111.1	Other	Other vertebrate	11.907	0.538	1	0	6658	18094	4	0.00342	12530	0.00383	-0.824	1.313	
HNF1B	MA0153.1	Helix-Turn-Helix	Homeo vertebrate	16.821	0.222	1	0	4107	20645	2	0.00187	6910	0.0023	-1.13	1.796	
Sox2	MA0143.1	Other	Alpha-Helix High	Mobility	Group	vertebrates	12.951	0.361	1	0	2809	21943	1	0.00117	3844	0.0016
PPARG	MA0066.1	Zinc-coordinating	Hormone-nuclear Receptor	vertebrates	20.365	0.523	0	1	62	24690	0	0	64	0.0000356	-1.416	0
NR1H2::RXRA	MA0115.1	Zinc-coordinating	Hormone-nuclear Receptor	vertebrates	27.878	0.445	0	1	99	24653	0	0	100	0.0000472	-1.421	0
Nkx3-2	MA0122.1	Helix-Turn-Helix	Homeo vertebrate	8.542	0.463	1	0	15425	9327	30	0.021	91612	0.0229	-1.489	0.473	
Pax4	MA0068.1	Helix-Turn-Helix	Homeo vertebrate	11.004	0.441	0	1	112	24640	0	0	113	0.0000942	-1.555	0	
EWSR1-FLI1	MA0149.1	Winged	Helix-Turn-Helix Ets	vertebrates	32.871	0.554	0	1	207	24545	0	0	209	0.000105	-1.591	0
REST	MA0138.2	Zinc-coordinating	BetaBetaAlpha-zinc finger	vertebrates	23.134	0.596	0	1	232	24520	0	0	242	0.000141	-1.719	0
EBF1	MA0154.1	Zipper-Type	Helix-Loop-Helix vertebrate	11.564	0.648	1	0	11288	13464	12	0.00933	39149	0.0109	-1.739	0.785	
ESR1	MA0112.2	Zinc-coordinating	Hormone-nuclear Receptor	vertebrates	13.563	0.594	0	1	345	24407	0	0	355	0.000197	-1.907	0
TAL1::TCF3	MA0091.1	Zipper-Type	Helix-Loop-Helix vertebrate	14.07	0.453	1	0	7491	17261	4	0.00373	14681	0.0049	-1.956	1.195	
NKX3-1	MA0124.1	Helix-Turn-Helix	Homeo vertebrate	11.127	0.179	1	0	12654	12098	21	0.0114	68823	0.0134	-1.974	0.671	

Egr1	MA0162.1	Zinc-coordinating	BetaBetaAlpha-zinc	finger	vertebrates		14.456	0.739	1	0	6364	18388	3	0.00256	12188	0.00373	-2.233	1.358
Foxd3	MA0041.1	Winged	Helix-Turn-Helix	Forkhead	vertebrates		12.945	0.223	1	0	11354	13398	15	0.014	49535	0.0165	-2.283	0.779
MAX	MA0058.1	Zipper-Type	Helix-Loop-Helix	vertebrate		12.685	0.571	1	0	7993	16759	4	0.00311	15900	0.00442	-2.305	1.13	
Ar	MA0007.1	Zinc-coordinating	Hormone-nuclear	Receptor	vertebrates		15.703	0.498	0	1	534	24218	0	0	554	0.000339	-2.327	0
TP53	MA0106.1	Zinc-coordinating	Loop-Sheet-Helix	vertebrate		26.239	0.603	0	1	8	24744	0	0	8	0.00000445	-2.33	0	
RORA_2	MA0072.1	Zinc-coordinating	Hormone-nuclear	Receptor	vertebrates		17.425	0.369	1	0	3571	21181	1	0.00109	5079	0.00198	-2.367	1.936
NFIL3	MA0025.1	Zipper-Type	Leucine	Zipper	vertebrates		14.139	0.265	1	0	6731	18021	4	0.00342	15828	0.00484	-2.382	1.302
TLX1::NFIC	MA0119.1	Helix-Turn-Helix::Other	Homeo::Nuclear	Factor	I-CCAAT-binding		vertebrate	19.665	0.598	0	1	1066	23686	0	0	1162	0.000452	-2.62
ARID3A	MA0151.1	Helix-Turn-Helix	Arid	vertebrate		9.896	0.062	1	0	16086	8666	51	0.0238	166235	0.0277	-2.743	0.431	
SRY	MA0084.1	Other	Alpha-Helix	High	Mobility		Group	vertebrates	9.193	0.238	1	0	14714	10038	31	0.0217	101929	0.0255
USF1	MA0093.1	Zipper-Type	Helix-Loop-Helix	vertebrate		11.29	0.643	1	0	9330	15422	5	0.00272	21993	0.00428	-2.774	0.976	
Tcfcp2l1	MA0145.1	Other	CP2	vertebrate		11.65	0.609	1	0	10591	14161	9	0.00979	32190	0.0125	-2.823	0.849	
HLF	MA0043.1	Zipper-Type	Leucine	Zipper	vertebrates		11.147	0.394	1	0	5508	19244	2	0.00187	9969	0.00332	-2.951	1.503
T	MA0009.1	Beta-Hairpin-Ribbon	T	vertebrate		17.863	0.452	0	1	1811	22941	0	0	2114	0.000646	-3.058	0	
PBX1	MA0070.1	Helix-Turn-Helix	Homeo	vertebrate		14.641	0.31	1	0	4107	20645	1	0.000933	6634	0.00221	-3.183	1.796	
RXR::RAR_DR5	MA0159.1	Zinc-coordinating	Hormone-nuclear	Receptor	vertebrates		16.004	0.535	0	1	1313	23439	0	0	1499	0.000708	-3.185	0
Myf	MA0055.1	Zipper-Type	Helix-Loop-Helix	vertebrate		15.914	0.609	1	0	10157	14595	7	0.00653	27519	0.00918	-3.195	0.891	
Myc	MA0147.1	Zipper-Type	Helix-Loop-Helix	vertebrate		11.157	0.686	1	0	8288	16464	4	0.00311	18476	0.00513	-3.276	1.094	
Pax5	MA0014.1	Helix-Turn-Helix	Homeo	vertebrate		12.432	0.575	0	1	1224	23528	0	0	1364	0.000758	-3.284	0	
FOXF2	MA0030.1	Winged	Helix-Turn-Helix	Forkhead	vertebrates		14.824	0.334	1	0	4097	20655	1	0.00109	6459	0.00251	-3.316	1.798
CREB1	MA0018.2	Zipper-Type	Leucine	Zipper	vertebrates		10.139	0.523	1	0	9204	15548	4	0.00249	19712	0.00438	-3.321	0.989
Arnt	MA0004.1	Zipper-Type	Helix-Loop-Helix	vertebrate		10.992	0.642	1	0	7052	17700	2	0.000933	13904	0.00232	-3.359	1.255	
znf143	MA0088.1	Zinc-coordinating	BetaBetaAlpha-zinc	finger	vertebrates		17.541	0.53	0	1	1301	23451	0	0	1473	0.000819	-3.401	0
Mycn	MA0104.2	Zipper-Type	Helix-Loop-Helix	vertebrate		11.104	0.699	1	0	8332	16420	4	0.00311	18928	0.00526	-3.433	1.089	
MEF2A	MA0052.1	Other	Alpha-Helix	MADS	vertebrates		15.709	0.179	1	0	7006	17746	3	0.00233	15631	0.00434	-3.537	1.262
Stat3	MA0144.1	Ig-fold	Stat	vertebrate		13.601	0.505	1	0	9883	14869	5	0.00389	23645	0.00657	-3.823	0.918	
ELK4	MA0076.1	Winged	Helix-Turn-Helix	Ets	vertebrates		14.123	0.583	1	0	6034	18718	1	0.0007	9342	0.00234	-3.937	1.411
Hand1::Tcfe2a	MA0092.1	Zipper-Type	Helix-Loop-Helix	vertebrate		10.144	0.507	1	0	13286	11466	14	0.0109	54768	0.0152	-4.054	0.622	
NFE2L2	MA0150.1	Zipper-Type	Leucine	Zipper	vertebrates		14.394	0.486	1	0	5635	19117	1	0.000855	8970	0.00274	-4.177	1.48
HIF1A::ARNT	MA0259.1	Zipper-Type	Helix-Loop-Helix	vertebrate		9.74	0.657	1	0	10914	13838	8	0.00497	37662	0.00837	-4.278	0.819	
Tal1::Gata1	MA0140.1	Zipper-Type	Helix-Loop-Helix	vertebrate		11.297	0.451	1	0	6212	18540	2	0.0028	11200	0.0056	-4.32	1.382	
MYC::MAX	MA0059.1	Zipper-Type	Helix-Loop-Helix	vertebrate		14.237	0.602	0	1	3436	21316	0	0	4535	0.00139	-4.344	0	
NR3C1	MA0113.1	Zinc-coordinating	Hormone-nuclear	Receptor	vertebrates		14.749	0.432	0	1	2522	22230	0	0	3161	0.00158	-4.625	0
NHLH1	MA0048.1	Zipper-Type	Helix-Loop-Helix	vertebrate		14.132	0.674	1	0	5783	18969	1	0.000933	9874	0.00329	-4.749	1.454	
NFYA	MA0060.1	Other	Alpha-Helix	NFY	CCAAT-binding		vertebrate	12.925	0.523	1	0	5865	18887	1	0.00124	9476	0.00421	-5.268
Pou5f1	MA0142.1	Helix-Turn-Helix	Homeo	vertebrate		14.808	0.308	0	1	3402	21350	0	0	5205	0.00217	-5.384	0	
GABPA	MA0062.2	Winged	Helix-Turn-Helix	Ets	vertebrates		13.335	0.647	1	0	9355	15397	2	0.00171	20927	0.0064	-6.723	0.973
FOXI1	MA0042.1	Winged	Helix-Turn-Helix	Forkhead	vertebrates		13.183	0.263	1	0	11680	13072	8	0.00746	49366	0.0165	-8.057	0.751
